# Temperature Measurement Timings and the Fever Detection Rate After Gastrointestinal Surgery: Retrospective Cross-Sectional Study

**DOI:** 10.2196/50585

**Published:** 2024-10-09

**Authors:** Shiqi Wang, Gang Ji, Xiangying Feng, Luguang Huang, Jialin Luo, Pengfei Yu, Jiyang Zheng, Bin Yang, Xiangjie Wang, Qingchuan Zhao

**Affiliations:** 1 Xijing Hospital of Digestive Diseases Xijing Hospital Fourth Military Medical University Xi'an China; 2 Medical Information Department Xijing Hospital Fourth Military Medical University Xi'an China

**Keywords:** fever, gastrointestinal surgery, temperature measurement, temperature, detection, gastrointestinal, cross-sectional study

## Abstract

**Background:**

Postoperative fever frequently indicates surgical complications and is commonly used to evaluate the efficacy of interventions against surgical stress. However, the presence of circadian rhythms in body temperature may compromise the accurate detection of fever.

**Objective:**

This study aimed to investigate the detection rate of fever under intermittent measurement.

**Methods:**

We retrospectively reviewed the clinical records of patients who underwent nonemergency gastrointestinal surgery between November 2020 and April 2021. Patients’ temperature data were continuously collected every 4 seconds using a wireless axillary thermometer, and fever was defined as a temperature exceeding 38 °C within a day. To simulate intermittent measurement in clinical practice, the body temperature at each hour was selected from the continuously collected temperature dataset. Considering that temperatures are measured multiple times per day, all possible measurement plans using intermittent measurement were composed by combining 1-24 time points from the 24-hour daily cycle. Fever was clinically diagnosed based on the temperature readings at the selected time points per day. The fever detection rates for each plan, with varying measurement times, were listed and ranked.

**Results:**

Based on the temperature data continuously collected by the thermometer, fever occurred in 60 (40.8%) of the 147 included patients within 3 days after surgery. Of the measurement plans that included 1-24 measurements daily, the fever detection rates ranged from 3.3% (2/60) to 85% (51/60). The highest detection rates and corresponding timings for measurement plans with 1, 2, 3, and 4 measurements daily were 38.3% (23/60; at 8 PM), 56.7% (34/60; at 3 AM and 7 or 8 PM), 65% (39/60; at 3 AM, 8 PM, and 10 or 11 PM), and 70% (42/60; at 12 AM, 3 AM, 8 PM, and 11 PM), respectively; and the lowest detection rates were 3.3% (2/60), 6.7% (4/60), 6.7% (4/60), and 8.3% (5/60), respectively. Although fever within 3 days after surgery was not correlated with an increased incidence of postoperative complications (5/60, 8.3% vs 6/87, 6.9%; *P*=.76), it was correlated with a longer hospital stay (median 7, IQR 6-9 days vs median 6, IQR 5-7 days; *P*<.001).

**Conclusions:**

The fever detection rate of the intermittent approach is determined by the timing and frequency of measurement. Measuring at randomly selected time points can miss many fever events after gastrointestinal surgery. However, we can improve the fever detection rate by optimizing the timing and frequency of measurement.

## Introduction

Fever commonly ensues following a diverse array of surgical interventions, including gastrointestinal procedures. Postoperative fever frequently stems from surgical stress and complicating factors [1,2]. In the context of gastrointestinal surgery, fever not only reflects elevated levels of surgical stress but may also signal the potential for complications, such as thrombosis, gastrointestinal leaks, intra-abdominal infections, and pulmonary infections [3-7]. In addition, fever manifestations are integral to the evaluation of targets and outcome measures for various Enhanced Recovery After Surgery (ERAS) interventions [8-14]. Therefore, precise fever detection is imperative for ensuring clinical safety and for the accurate appraisal of treatment efficacy. Time and accurate detection of fever promotes the early identification of patients at heightened risk for complications and supports the precise assessment of the efficacy of perioperative stress mitigation strategies.

To detect postoperative fever, patient temperatures are routinely measured at intervals of several hours against a predetermined threshold. A majority of health care facilities use a body temperature over 38 °C as the criterion for fever diagnosis [15]. This threshold is also commonly used in many clinical studies [5,16,17]. Nevertheless, there is often a failure to account for the timing of temperature measurements, which is pivotal since body temperature naturally oscillates, reaching a nadir at 6 AM and peaking between 4 PM and 6 PM [18]. As such, disparate measurement timings can result in substantial variance in the detected rates of fever within study cohorts. The question arises as to the optimal timing for temperature checks to identify fever. To date, neither a consensus nor guidelines exist to address this issue. Furthermore, the timing of temperature measurement is rarely specified in the literature on postoperative fever, with common practices including twice daily [17], during morning rounds [16], every 8 hours [19,20], or not report at all in certain studies [5,9,17,21-25]. This inconsistency in monitoring may overlook many febrile episodes, impeding the ability to evaluate the relationship between fever and surgical outcomes, as well as the effectiveness of perioperative antistress interventions.

Enhancing the frequency of temperature measurements could potentially improve fever detection rates; however, the practicality of such a method is low, incurring a substantial increase in medical workload for relatively little gain. Research has explored the use of wireless sensors for continuous real-time monitoring of patients’ body temperatures. These sensors, transmitting temperature data to a central processor at frequent intervals, offer a more precise reflection of temperature fluctuations [26,27], which can facilitate prompt identification of postoperative complications [28]. Nonetheless, the functionality of these sensors is heavily dependent on reliable local network infrastructure and related devices. In settings bereft of sensor technology, alternative strategies must be considered. Harding et al [29] noted that fever patterns correspond to the diurnal variation in body temperature, peaking and troughing at consistent times, with nighttime fever incidence in the emergency department exceeding morning rates by a factor of 2.5. Such findings imply that adjusting measurement timings could improve fever detection rates. However, the optimal intervals and frequency of temperature assessments for maximal fever detection efficacy are yet to be determined. This study gathered data on the hour from a continuous temperature dataset and systematically constructed various hypothetical measurement schedules, subsequently evaluating the fever detection rate of each regimen to ascertain the most effective timing for temperature checks.

## Methods

### Study Design and Participants

This was a retrospective cross-sectional study. Between November 29, 2020, and April 1, 2021, consecutive patients who were aged 18 years or older and underwent nonemergency gastrointestinal surgery were included. Patients who took immunosuppressive drugs within 4 weeks before surgery, who had used antibiotics or antipyretic analgesics in the week before admission, and who were pregnant were excluded. To avoid the interference of nonsurgical fever, we also excluded patients who presented with fever before surgery. Patients whose temperature data were missing for any reason were also excluded. Demographic characteristics, surgical types, length of hospital stay, and in-hospital complications were collected.

### Ethical Considerations

This study was approved by the Ethics Committee of the First Affiliated Hospital of Air Force Medical University (approved KY20222271-C-1). The patients’ data have been anonymized. As a retrospective study, there was no compensation, and the requirement for informed consent was waived.

### Body Temperature Measurement

Body temperature was continuously measured every 4 seconds by a wireless axillary thermometer (iThermonitor; Raiing Medical Company). The measurement accuracy of the sensor is 0.01 °C, and the readings are consistent with those of mercury thermometers [30]. On admission, a hypoallergenic adhesive patch (Raiing Medical Company) was used to securely position the iThermonitor in the shaved axilla of the patient. The temperature data were transmitted to a repeater through low-energy Bluetooth and then transferred to a central workstation, where the data were saved on an electronic monitoring panel. A detailed description of the technical parameters of the iThermonitor can be found in Multimedia Appendix 1. The temperature data on the day of surgery were dismissed, and the temperature data of the next 3 days were retrieved and named the first-, second-, and third-day temperatures.

### End Points

Taking into consideration previous research reports and consensus outcomes, fever was defined as a body temperature that exceeded 38 ℃ [5,15-17]. Based on the definition of fever and the data conscientiously collected by the sensors, the fever incidence on each day of the first 3 days and the total fever incidence of the first 3 days were investigated.

Patients were divided into a fever group and a nonfever group, and the clinical outcomes were compared between the 2 groups. The correlation between fever on the first day and fever in the next 2 days was investigated.

### Intermittent Measurement Simulation

In the simulated clinical temperature measurement analysis, we included all patients who were determined to have a fever based on sensor temperature data. In clinical practice, for ease of implementation and documentation, temperature measurements are typically taken on the hour. Therefore, the temperature data for every hour were selected from the consecutively collected dataset.

A brute force strategy was used to list the fever detection rate of every possible measurement plan with varied measurement time points per day. The simulated intermittent measurement plans, where every possible temperature measurement plan in clinical practice, including 1 to 24 time points, were composed of n time points from the 24 hours, such as C (24, 1), C (24, 2), C (24, 3), C (24, 4), C (24, 6), C (24, 8), C (24, 12), and C (24, 24). If the temperature data at any time points included in the temperature measurement plan exceed 38 °C, it was considered that the clinical temperature measurement plan has detected a fever.

For example, C (24, 2) means diagnosing fever based on temperatures at any 2 time points in 24 hours, such as 12 AM and 1 AM, 12 AM and 2 AM, or 12 AM and 3 AM. The total number of combinations of C (24, 2) was 276. Fever was clinically diagnosed based on the temperature at the selected time points, and the fever detection rates of every plan with varied measurement times were listed and ranked.

### Statistical Analysis

Temperature data processing, including the combination of the measurement timings, calculation of fever incidence, and calculation of fever detection rate, was managed by Python (version 3.7.3; Python Software Foundation), pandas (version 1.1.3; Pandas Development Team), and NumPy (version 1.21.6; NumPy Community). The statistical analysis was conducted by SPSS 22.0 (IBM Corp). Categorical data are reported as numbers with proportions, and quantitative data are reported as the mean with SD or, where appropriate, as the median with an IQR. Categorical data were compared using the chi-square test or Fisher exact test, where appropriate. For continuous data, the Student *t* test (2-tailed) or Mann-Whitney *U* test was used. A 2-sided *P* value of <.05 was considered statistically significant. Because of the exploratory nature of this survey, the sample size calculation was not performed.

## Results

### Patient Characteristics and Clinical Outcomes

A total of 147 patients who underwent gastrointestinal surgery were included. All the patients had complete temperature data within 3 days after surgery, and no missing values needed to be processed. Temperature data that were continuously collected by the sensor were used. Fever was detected in a total of 40.8% (60/147) patients within 3 days after surgery. Table 1 shows the demographic characteristics, surgical types, length of hospital stay after surgery, and in-hospital complications. The median age of the patients was 60 (IQR 53-67) years, and 62.6% (92/147) of the patients were male. The median length of hospital stay was 6 (IQR 5-8) days. Compared with the patients without fever within 3 days after surgery, patients with fever experienced a longer length of hospital stay (median 7, IQR 6-9 days vs median 6, IQR 5-7 days; *P*<.001). No significant difference in the postoperative complication rate was found between patients with and without fever within the 3 days (5/60, 8.3% vs 6/87, 6.9%; *P*=.76).

**Table 1:** Patient characteristics.

Characteristics		All patients (N=147)	Fever (n=60)	No fever (n=87)	*P* value	
**Age (years), median (IQR)**		60 (53-67)	60.5 (56-66)	58 (50-69)	.30^a^	
**Sex (male), n (%)**		92 (62.6)	36 (60)	56 (64.4)	.59^b^	
**Comorbidity, n (%)**						
	Hypertension	28 (19)	12 (20)	14 (16.1)	.54^b^	
	Diabetes	11 (7.5)	4 (6.8)	7 (8)	>.99^c^	
	Coronary artery disease	12 (8.2)	5 (8.3)	7 (8)	>.99^c^	
**Laparoscopic surgery, n (%)**		81 (55.1)	34 (56.7)	47 (54)	.75^b^	
**Surgery types, n (%)**						.15^c^
	Esophagectomy	5 (3.4)	2 (3.3)	3 (3.4)		
	Gastrectomy	60 (40.8)	32 (53.3)	28 (32.2)		
	Colorectal resection	72 (49)	24 (40)	48 (55.2)		
	Small intestinal resection	7 (4.8)	2 (3.3)	5 (5.7)		
	Pancreaticoduodenectomy	2 (1.4)	0 (0)	5 (5.7)		
	Pancreatectomy	1 (0.7)	0 (0)	1 (1.1)		
**Length of hospital stay (days), median (IQR)**		6 (5-8)	7 (6-9)	6 (5-7)	<.001^d^	
**Complications, n (%)**		11 (7.5)	5 (8.3)	6 (6.9)	.76^c^	
	Pneumonia	4 (2.7)	2 (3.3)	2 (2.3)		
	Intestinal obstruction	4 (2.7)	2 (3.3)	2 (2.3)		
	Leakage	3 (2)	1 (1.7)	2 (2.3)		
	Incision dehiscence	2 (1.4)	1 (1.7)	1 (1.1)		

^a^Student *t* test (2-tailed).

^b^Chi-square test.

^c^Fisher exact test.

^d^Mann-Whitney *U* test.

### Fluctuation of Body Temperature and Fever Detection Rate

**Figure 1:**
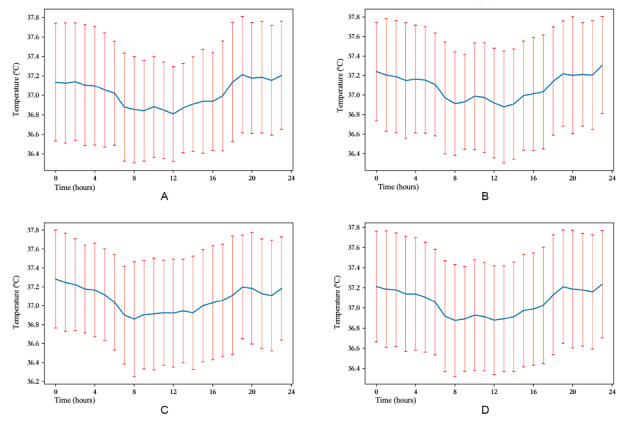
Body temperature curve after gastrointestinal surgery. Body temperature fluctuations on the (A) first, (B) second, and (C) third days after surgery. The average body temperatures in the first 3 days after surgery were 37.02 °C, 37.08 °C, and 37.06 °C, respectively. (D) Average body temperature within 24 hours. The average body temperature bottomed out at 8 AM and peaked at 11 PM.

The fluctuations in body temperature on the first, second, and third days after surgery are shown in Figure 1A-C. The mean body temperature ranged from 36.88 °C to 37.24 °C, and the mean body temperatures in the first 3 days after surgery were 37.02 °C, 37.08 °C, and 37.06 °C, respectively.

**Figure 2:**
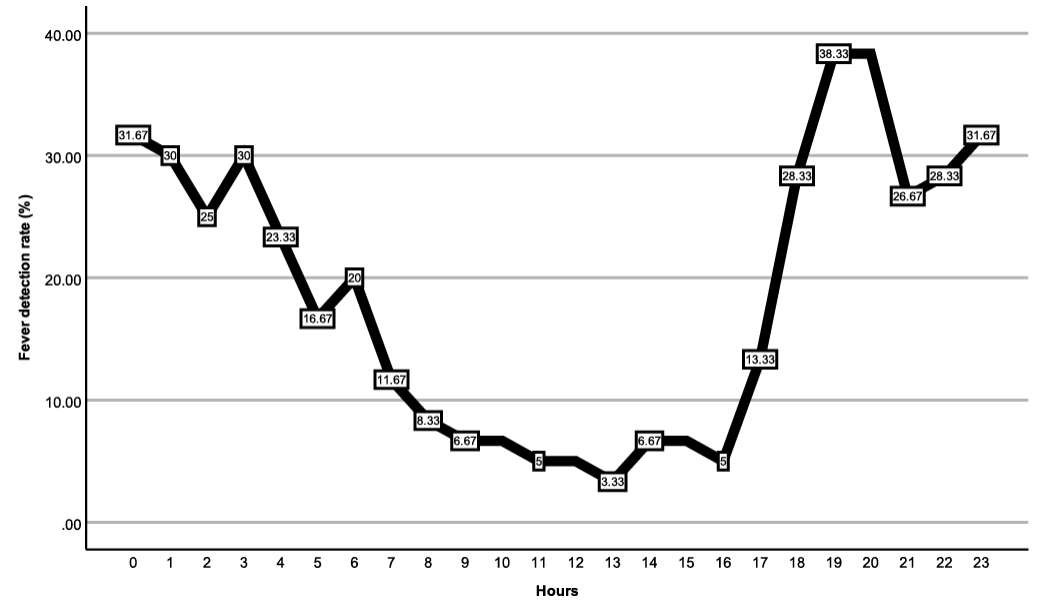
Fever detection rate on each hour. The vertical axis represents the fever detection rate, and the abscissa axis represents the hours. The fever detection rate peaked at 38.3% at 7 PM and 8 PM and reached a nadir of 3.3% at 1 PM.

The average body temperature in 1 day is shown in Figure 1D**,** and the body temperature varied throughout the day, with its nadir at 8 AM and its zenith at 11 PM. Figure 2 shows the fever detection rate by each hour. Within 24 hours, 38.3% (23/60) fever was detected by taking measures at 7 PM and 8 PM, and only 3.3% (2/60) fever was detected by taking measures at 1 PM.

### Fever Detection Rate and Measurement Times

**Figure 3:**
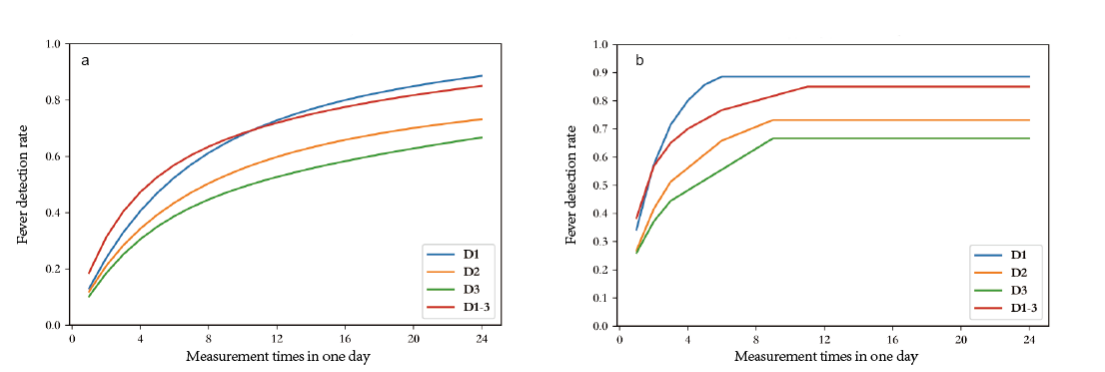
Correlation between fever detection rate and measurement times. The vertical axis represents the fever detection rate, and the abscissa axis represents the measurement times. The blue (D1), orange (D2), and green (D3) lines show the fever detection rates on the first, second, and third days after surgery, respectively. The red line (D1-3) shows the fever detection rate within 3 days. (A) The averaged fever detection rates with varied time points. (B) The highest detection rates with varied time points.

Using intermittently collected temperature data on the hour, varied measurement plans were constructed and demonstrated. The highest and lowest detection rates and the measurement timings for the highest detection rate are shown in Table 2. For the 1–time point model C (24, 1), meaning fever was diagnosed by the temperature data at 1 time point per day, the fever detection rate ranged from 3.3% (2/60) to 38.3% (23/60). In the 2–time point model C (24, 2), meaning fever was diagnosed by the temperature data at 2 time points per day, the fever detection rate ranged from 6.7% (4/60) to 56.7% (34/60). For the C (24, 3), C (24, 4), and C (24, 6) models, the fever detection rate ranged from 6.7% (4/60) to 65% (39/60), from 8.3% (5/60) to 70% (42/60), and from 11.7% (7/60) to 76.7% (46/60), respectively. When the measurement frequency was increased to hourly, the detection rate gradually reached a plateau of 85% (51/60) (Figure 3A).

**Table 2:** Fever detection rates of body temperature measurement plans with varied measurement timings.

Measurement timings			Fever detection rate^b^ (%, 95% CI)			Measurement plans with the highest detection rate
		Lowest		Highest		
**Measurement plans ^a^**						
	C (24, 1)	3.3 (0.4-6.2)		38.3 (30.5-46.2)	7 or 8 PM	
	C (24, 2)	6.7 (2.6-10.7)		56.7 (48.7-64.7)	3 AM and 7 or 8 PM	
	C (24, 3)	6.7 (2.6-10.7)		65.0 (57.3-72.7)	3 AM, 8 PM, and 10 or11 PM	
	C (24, 4)	8.3 (3.9-12.8)		70.0 (62.6-77.4)	12 AM, 3 AM, 8 PM, and 11 PM	
	C (24, 6)	11.7 (6.5-12.9)		76.7 (69.8, 83.5)	12 AM, 3 AM, 6 AM, 4 PM, 8 PM, and 11 PM	
	C (24, 8)	18.3 (12.1-24.6)		80.0 (73.5-86.5)	12 AM, 1 AM, 3 AM, 5 AM, 6 AM, 4 PM, 8 PM, 11 PM, etc (n=26)	
	C (24,24)	85.0 (79.2-90.8)		85.0 (79.2-90.8)	—^c^	
**Plans in our ward**						
	Plan A	43.3 (40.3-56.4)		43.3 (40.3-56.4)	6 AM and 6 PM	
	Plan B	48.3 (40.3-56.4)		48.3 (40.3-56.4)	6 AM, 10 AM, 2 PM, and 6 PM	
	Plan C	58.3 (50.4-66.3)		58.3 (50.4-66.3)	6 AM, 10 AM, 2 PM, 6 PM, 10 PM, and 2 AM	

^a^C (24, r), selecting r time points from the 24 hours in 1 day.

^b^Fever detection rate = (detected number of patients with fever) / (all patients with fever) within 3 days after surgery.

^c^Not applicable.

### Fever Detection Rate and Measurement Timings

The detection rate of the intermittent approach is influenced by the measurement timings. Table 2 shows that the corresponding time points of the top detection rates were distributed throughout the nighttime. For example, at 7 or 8 PM for C (24, 1); 3 AM and 7 or 8 PM for C (24, 2); 3AM, 8 PM, and 10 or 11 PM for C (24, 3); and 12 AM, 3 AM, 8 PM, and 11 PM for C (24, 4), the detection rate also reached a plateau by taking measures at fewer specific time points (Figure 3B). For example, an 85% (51/60) detection rate can also be achieved by using 11 time points: 12 AM, 1 AM, 3 AM, 5 AM, 6 AM, 8 AM, 4 PM, 5 PM, 8 PM, 9 PM, and 11 PM (see row C (24, 11) in Multimedia Appendix 2). However, this is also cumbersome in real clinical work.

In our ward, according to the nursing grade, postoperative temperature are measured with 3 plans: plan A (6 AM and 6 PM), plan B (6 AM, 10 AM, 2 PM, and 6 PM), and plan C (6 AM, 10 AM, 2 PM, 6 PM, 10 PM, and 2 AM). Based on the continuously collected data, the fever detection rate of these plans were 43.3% (26/60), 48.3% (29/60), and 58.3% (35/60), respectively. The optimal detection rate of plans with the same measurement times were 56.7% (34/60), 70% (42/60), and 76.7% (46/60), respectively (Table 2).

## Discussion

### Principal Findings

This is the first study to investigate fever detection rates by intermittent temperature measurements. In this study, every possible intermittent measurement plan with varied measurement timings was constructed, and the corresponding fever detection rates were calculated. The results showed that fever was less frequently detected by medical staff than by the sensors, and the upper limit of detection rates by intermittent measurement was 85% (51/60) when body temperature was measured every hour. For measurement plans with varied daily frequencies, we can improve the detection rates by adjusting the measurement timings.

### Limitations

While our findings have important implications, we acknowledge several limitations to our study. First, this is a small-sample retrospective study; as such, its results may be subject to bias. Second, we included patients who had undergone gastrointestinal surgery; it remains to be verified whether patients who have undergone other types of surgery also exhibit similar postoperative body temperature characteristics, which would require validation in other patient cohorts. Third, the detection rates were calculated based on the assumption that temperature was measured on the hour. There may be better time points at which fever detection is the highest. However, for convenience, body temperature is usually measured during the hour of clinical work, and our assumption is consistent with clinical practice. Fourth, previous studies and our research both indicate that the incidence of febrile events decreases as the duration of hospitalization increases [31-33]. Given that our study had a small sample size, and febrile events became infrequent after 3 days, it was challenging to discern the differences across various temperature monitoring schedules. Therefore, we chose to analyze the time period during which the occurrence of febrile events was higher, and only body temperature within the first 3 days after surgery was recorded. Whether the selected time points that were determined in this study are applicable after 3 days remains to be determined in further studies. Fifth, like most studies, we used a fixed threshold to define fever at different times. However, considering the variability of body temperature, it might be more reasonable to use a floating threshold to determine whether a patient is having a fever at different times. For instance, whether a body temperature exceeding 37.5 ℃ after waking up, or exceeding 37 ℃, should also be considered an abnormal state. Nonetheless, we currently lack a more rational method to define fever. Moreover, this issue goes beyond the interpretive scope of this study and requires further exploration in future research. In addition, body temperature is influenced by age, sex, and even weather [34]. However, stratified analysis was not performed since it is not practical to do so in the ward to define fever by varied levels.

### Comparison with Previous Work

Consistent with a previous study which found that the proportion of patients with fever increased 2.4 to 3.6 times from morning to evening [29], we also observed that the timings of the measurement plans with the highest detection rates were predominantly at night. Moreover, as Figure 2 illustrates, the discrepancy between fever detection rates during the day and at night was more pronounced (2/60, 38.3% at 7 or 8 PM vs 2/60, 3.3% at 1 PM). This can be explained by the circadian rhythm of human body temperature. It is well known that the body temperature fluctuates throughout the day [18], with potential fluctuations of up to 1 °C within a single day [35]. In our study, we also found that body temperature exhibited rhythmic variations in patients who underwent gastrointestinal surgery. Figure 1D demonstrates that patients’ body temperatures after surgery tend to be higher at night and lower during daylight, with the minimum recorded at 8 AM and the maximum at 11 PM. Hence, assessing fevers using temperature readings taken at various times throughout the day can lead to substantial discrepancies in conclusions.

Unfortunately, the timing of temperature monitoring is often neglected in both clinical research and practical settings. Notably, even in medical students’ textbooks, there is no clear protocol for monitoring body temperature during the perioperative period. Medical centers tend to formulate postoperative temperature monitoring protocols based on customary local practices rather than standardized guidelines. This study demonstrates that our hospital’s long-standing protocol has failed to effectively identify postoperative fever events. As indicated by Table 2, conducting as many as 6 temperature measurements daily only detected 58.3% (35/60) of patients with fever. If we adjust the temperature measurement times to 3 AM and 7 or 8 PM, 2 daily measurements could still identify 56.7% (34/60) of the cases. Optimizing the schedule to include checks at 12 AM, 3 AM, 6 AM, 4 PM, 8 PM, and 11 PM could improve fever detection rates to 76.7% (46/60).

As with our usual practice, some studies on the clinical significance of fever are typically measured only at a few unreported times of the day [16,17,19,20], while other studies do not show the timing of measurement [6,9,17,21-25]. Assuming body temperature is measured per hour, there would be 24 time points per day, resulting in a vast array of measurement schedules. Given the wide range of detection rates among the numerous measurement schedules, many patients with fever might go unidentified when measurements are taken at randomly selected times. Since fevers cannot be accurately detected, the interpretation of the clinical significance of postoperative fever may also be biased. In addition, we also see some clinical studies that consider fever as an outcome of the intervention, especially those related to ERAS strategies for perioperative stress control [8-14]. In these studies, the timing and frequency of temperature measurements are not reported either. If consistency of the timing of temperature measurements is not considered when assessing fever, biases are likely to occur when evaluating the efficacy of the respective clinical interventions. Therefore, we may consider including recommendations on the timing of temperature measurements in ERAS-related guidelines.

Multimedia Appendix 2 lists the optimal measurement schedules that achieve the highest detection rates, varying from once to 23 times per day. By aligning these schedules with the routine practices of local hospitals, clinicians can formulate more precise thermometric protocols. For instance, a tridaily measurement regimen might entail taking temperatures at 3 AM, 8 PM, and 10 or 11 PM, as specified in row C (24, 3) in Table 2. It is essential to recognize, however, that these proposed times are flexible rather than absolute mandates for fever surveillance. In clinical practice, temperatures will also be measured at any necessary time. Given the symptoms that accompany fever, it is reasonable to measure temperature at suggested times as well as when needed.

What is the clinical significance of detecting postoperative fever, especially fever that occurs in the early days following surgery? Many studies have investigated the correlation between postoperative fever and infection. Most have found that postoperative fever is a marker of infection with very low specificity and sensitivity [2,5]. Some researchers have reported that among patients who developed fever after abdominal surgery, only 2% had positive blood cultures [5]. Among patients with fever following orthopedic surgeries, the positive finding rates for chest x-rays, urinalysis, urine cultures, and blood cultures were 0.3%, 28.5%, 10.9%, and 3.5%, respectively. Such low cost-effectiveness has led some researchers to question the use of postoperative temperature measurement [36]. One study even instructed the clinical team responsible for patient care to remain unaware of the patient’s body temperature and required clinical decisions to be made without looking at temperature data. This study reported a positive predictive value of merely 8% for fever as an indicator of infection, suggesting the potential abandonment of routine temperature measurements [17]. Our findings align with these observations, demonstrating an insignificant link between fevers within the first 3 postoperative days and the onset of complications.

Although the prevalence of postoperative fever may not require immediate imaging or bacteriological assessments, it is inadvisable to ignore it and leave patients to manage the condition without support. The risks posed by postoperative fever extend beyond infection. Postoperative fever is also associated with the release of inflammatory mediators in the absence of infection. This study indicates that fever during the early postoperative days has a positive correlation with prolonged hospital stays (with a median of 7, IQR 6-9 days, compared with a median of 6, IQR 5-7 days). The longer hospitalization might be attributable to surgical stress, as a fever following surgery may arise from inflammation and tissue damage [37], suggesting that patients enduring pronounced surgical stress may need additional recovery time. Monitoring for postoperative fever is crucial in evaluating the magnitude of surgical stress and the efficacy of interventions to mitigate it. Fever, as a surgical stressor, constitutes a postoperative adverse event and an unpleasant experience that necessitates closer nursing attention. Considering the benign nature of early fever, there might be 2 approaches, refraining from intervention and allowing the fever to subside on its own, or providing necessary medical care, such as pain management [38], physical cooling, physical examinations, and psychological comfort, to facilitate the recovery process. If we opt to take some action, routine ward rounds could be considered during peak fever times, such as between 7 PM and 8 PM.

### Conclusions

In conclusion, reliance on traditional, arbitrary temperature measurement can lead to the oversight of numerous febrile episodes. From the standpoint of both clinical safety concerning fevers and the interpretability of clinical research, it is necessary to improve the detection rate of postoperative febrile events. Even in medical settings where continuous temperature monitoring sensors are unavailable, adjusting the timing for measuring temperatures to the nighttime can substantially improve the detection of febrile events. Postoperative body temperature monitoring protocols can be revised in accordance with the working habits of local hospitals. In addition, to facilitate more precise assessments of study outcomes, future research examining postoperative fevers should consider detailing the timing of temperature recordings in their reports.

## Appendix

Multimedia Appendix 1 Description of iThermonitor wireless temperature monitoring technology.

Multimedia Appendix 2 Fever detection rates of measurement plans with varied measurement timings.

## Abbreviations

ERAS: Enhanced Recovery After Surgery
