# Supplementary material for: Temperature Measurement Timings and the Fever Detection Rate After Gastrointestinal Surgery: Retrospective Cross-Sectional Study
Source: Interact J Med Res. 2024 Oct 9;13:e50585. doi: 10.2196/50585 (PMC11499718; doi:10.2196/50585)

**Description of iThermonitor® wireless temperature monitoring technology**

As shown in the figure, the axillary artery of the human body is deep in the axilla, behind the pectoralis major and minor muscles, which provides abundant arterial blood flow for the upper limbs and axilla, while the axillary artery blood temperature can reach or approach the core body temperature.


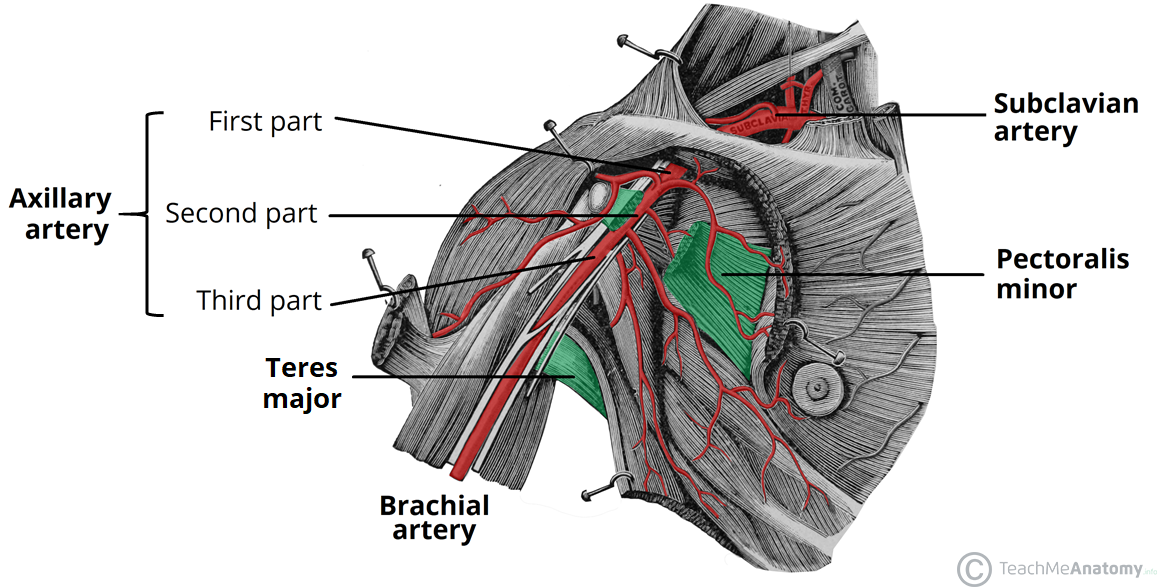


Wireless Body Temperature

Sensor

In a warm and stable environment, such as a hospital, the temperature measured near the axillary artery after adduction and clamping of one’s arm for a period of time can be similar to core body temperature. ^1,2,3.^ In contrast, the axillary temperature measured at random is only approximate to the skin temperature and is often lower than the core body temperature. To sum up, there is a certain relationship between armpit heat flow and core body temperature, and the relationship between the measured axillary temperature and core body temperature largely depends on the measurement technology and clinical environment.

The wireless temperature sensor iThermonitor® used in this study is an innovative continuous temperature monitoring device that uses a high-precision thermistor probe attached to the axillary root (lateral body surface of the axillary artery), a model algorithm based on a large number of experimental data accumulation, and computer programs to predict and correct body temperature. The parameter values of the sensor come from the raw measurement data which go through the noise reduction algorithm (filtered and anti-interfered) , an innovative algorithm based on a large sample of data in operating room environment, such as room temperature, human bladder, esophageal, axillary temperature experimental data from different parts of human body structure. In the clinical validation studies, compared to human esophageal temperature, iThermonitor reached 0.06 ^4^ ℃ ~ 0.14 ℃ ^5^ average difference. When body temperature is monitored by the wireless temperature sensor iThermonitor®, the arms of subjects should be closed for at least the first 8 minutes, providing good measuring conditions.


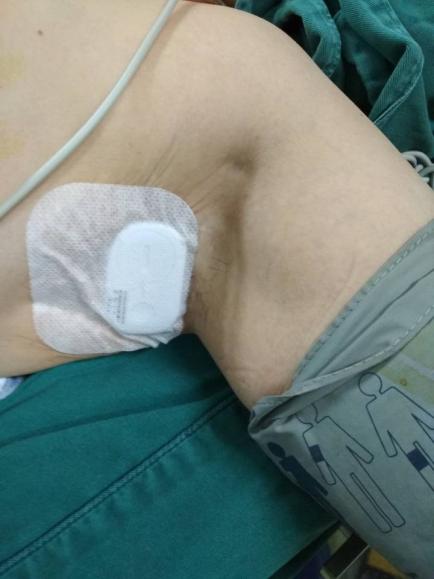

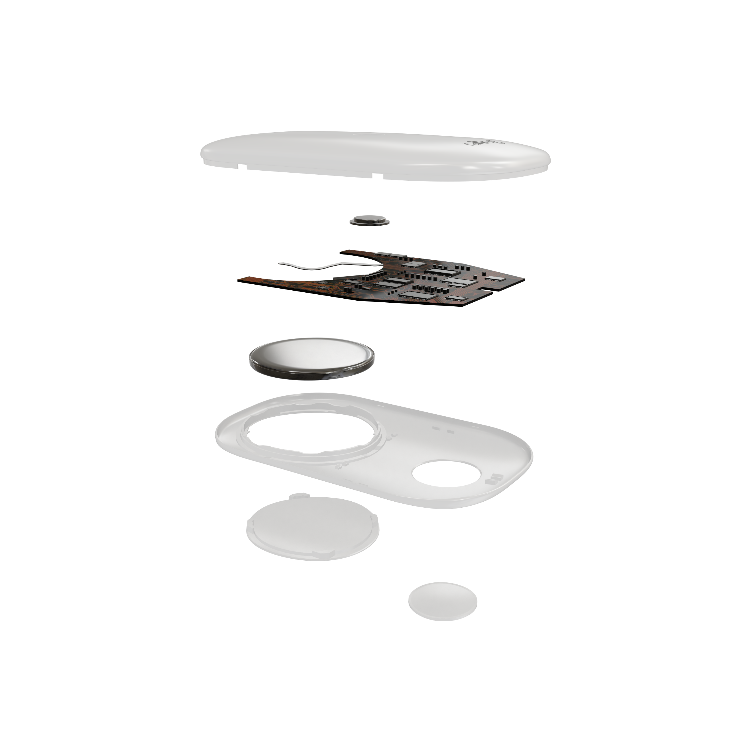


*1.Langham GE, Maheshwari A, Contrera K, You J, Mascha E, Sessler DI: Noninvasive temperature monitoring in postanesthesia care units. Anesthesiology 2009; 111: 90-6*

*2.Giuffre M, Heidenreich T, Carney-Gersten P, Dorsch JA, Heidenreich E: The relationship between axillary and core body temperature measurements. Appl Nurs Res 1990; 3: 52-5*

*3.Lodha R, Mukerji N, Sinha N, Pandey RM, Jain Y: Is axillary temperature an appropriate surrogate for core temperature? Indian J Pediatr 2000; 67: 571-4crystal thermometers. Anesthesiology 1997; 86: 603-12.*

*4. 蔡晶晶，吴薇薇，朱涛. 无线体温传感器监测日间手术术中核心体温的可行性: 前瞻性诊断试验. [J]. 协和医学杂志，2019，10（6）：45-48.*

*5. Lijian Pei, MD, Yuguang Huang, MD, Guangmei Mao, PhD, MPH, and Daniel I. Sessler, MD. Axillary Temperature, as Recorded by the iThermonitor WT701, Well Represents Core Temperature in Adults Having Noncardiac Surgery. [J]. Anesthesia & Analgesia. March 2018 -Volume 126 - Issue 3 - p 833–838.*

**Description of CareClever® wireless body temperature monitoring system**

**The working principle of the CareClever® wireless temperature monitoring system is outlined as follows:**

**Sensor Attachment and Data Collection:**

- Patients wear the iThermonitor® wireless temperature sensor on their bodies using medical adhesive patches.
- The sensor continuously monitors and generates temperature data at a frequency of every 4 seconds.

**Data Wireless Transmission (Low Energy Bluetooth):**

- The iThermonitor® wireless temperature sensor utilizes low energy Bluetooth (BLE) technology to send real-time temperature data to a relay device.
- The low energy Bluetooth ensures that the sensor can operate for long periods without the need for frequent battery replacements.

**Data Conversion and Transmission by the Relay Device:**

- The relay device, fixed next to the bed, receives Bluetooth signals from the sensor.
- The device converts the Bluetooth signals into Wi-Fi signals for data transmission over a wider network range.
- The relay device sends the temperature data to a central workstation via Wi-Fi.

**Data Processing and Display at the Central Workstation:**

- The central workstation is a device with a large screen, which receives, processes, and displays the temperature data from the relay.
- Nurses can view patients' real-time temperature data, historical records, and other related information on the central workstation.

**Alarm System:**

- The system features an over-temperature alarm function that triggers an alert when a patient's temperature exceeds the preset safe range.
- Technical alarms are also triggered in cases of poor wearing quality, disconnected sensors, or low battery, ensuring nurses can address these issues promptly.

**Operating Duration and Power Supply:**

The iThermonitor® wireless temperature sensor can operate continuously for at least 30 days, reducing the frequency of battery replacements or sensor reattachment.

Both the relay device and the central workstation operate on electricity, ensuring system stability and continuous operability.

The entire system utilizes wireless technology to achieve real-time, continuous monitoring of temperature data, improving the efficiency and accuracy of medical care while reducing the workload of healthcare professionals.

**Technology roadmap of CareClever® wireless body temperature monitoring system:**


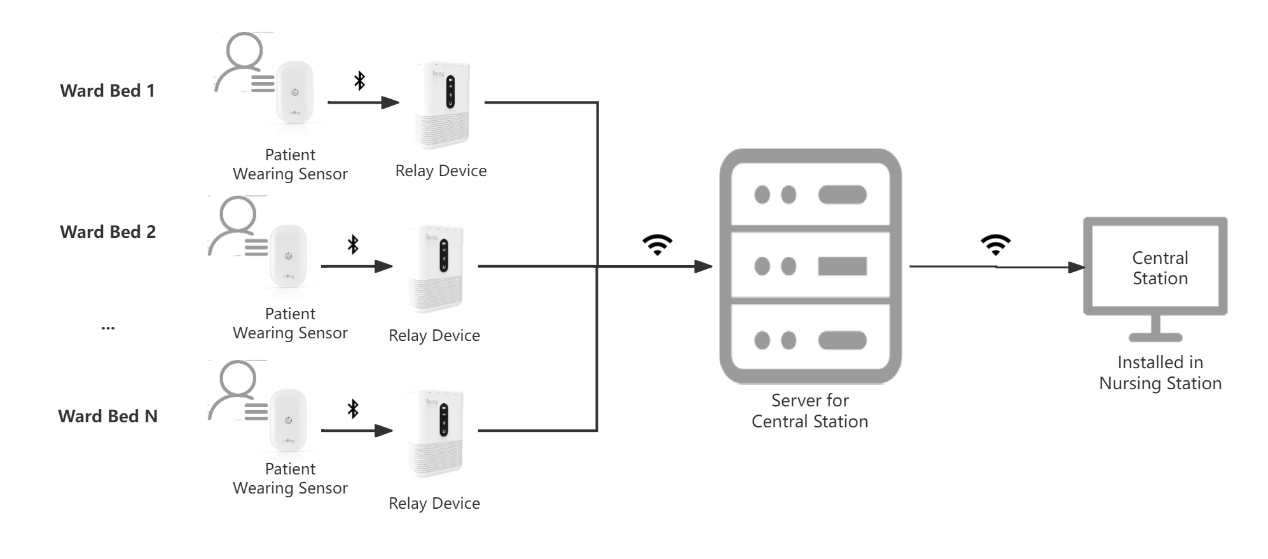

Supplement: Multimedia Appendix 1 [file ijmr_v13i1e50585_app1.docx]
