# Supplementary material for: Temperature Measurement Timings and the Fever Detection Rate After Gastrointestinal Surgery: Retrospective Cross-Sectional Study
Source: Interact J Med Res. 2024 Oct 9;13:e50585. doi: 10.2196/50585 (PMC11499718; doi:10.2196/50585)
Supplement: Multimedia Appendix 2 [file ijmr_v13i1e50585_app2.docx]

**Multimedia Appendix 1. Fever detection rates of measurement plans with varied measurement timings.**

| **Measurement plans** | **Detection rate (%)** | | | | | | | | **Measurement plans with the highest detection rate within D1-3** |
| --- | --- | --- | --- | --- | --- | --- | --- | --- | --- |
|  | **Lowest** | | | | **Highest** | | | |  |
|  | **D1** | **D2** | **D3** | **D1-3** | **D1** | **D2** | **D3** | **D1-3** |  |
| **C(24,1)** | 0 | 2.44 | 0 | 3.33 | 34.29 | 26.83 | 25.93 | 38.33 | 19:00/20:00 |
| **C(24,2)** | 0 | 2.44 | 0 | 6.67 | 57.14 | 41.46 | 37.04 | 56.67 | 3:00, 19:00/20:00 |
| **C(24,3)** | 0 | 2.44 | 0 | 6.67 | 71.43 | 51.22 | 44.44 | 65.00 | 3:00, 20:00, 22:00/23:00 |
| **C(24,4)** | 0 | 4.88 | 3.70 | 8.33 | 80.00 | 56.10 | 48.15 | 70.0 | 0:00, 3:00, 20:00, 23:00 |
| **C(24,5)** | 0 | 7.32 | 3.70 | 10.00 | 85.71 | 60.98 | 51.85 | 73.33 | 0:00, 1:00, 19:00/21:00, 20:00, 23:00 |
| **C(24,6)** | 0 | 7.32 | 11.11 | 11.67 | 88.57 | 65.85 | 55.56 | 76.67 | 0:00, 3:00, 6:00, 16:00, 20:00, 23:00 |
| **C(24,7)** | 0 | 7.32 | 11.11 | 15.00 | 88.57 | 68.29 | 59.26 | 78.33 | 0:00, 1:00, 3:00, 6:00, 16:00, 20:00, 23:00 et al. n=9 |
| **C(24,8)** | 2.86 | 7.32 | 11.11 | 18.33 | 88.57 | 70.73 | 62.96 | 80.00 | 0:00, 1:00, 3:00, 5:00, 6:00, 16:00, 20:00, 23:00 et al. n=26 |
| **C(24,9)** | 2.86 | 9.76 | 14.81 | 20.00 | 88.57 | 73.17 | 66.67 | 81.67 | 0:00, 1:00, 3:00, 5:00, 6:00, 8:00, 16:00, 20:00, 23:00 et al. n=34 |
| **C(24,10)** | 5.71 | 14.63 | 14.81 | 28.33 | 88.57 | 73.17 | 66.67 | 83.33 | 0:00, 1:00, 3:00, 5:00, 6:00, 8:00, 16:00, 17:00, 20:00, 23:00 et al. n=21 |
| **C(24,11)** | 14.29 | 19.51 | 18.52 | 36.67 | 88.57 | 73.17 | 66.67 | 85.00 | 0:00, 1:00, 3:00, 5:00, 6:00, 8:00, 16:00, 17:00, 20:00, 21:00, 23:00 et al. n=5 |
| **C(24,12)** | 22.86 | 24.39 | 22.22 | 41.67 | 88.57 | 73.17 | 66.67 | 85.00 | 0:00, 1:00, 2:00, 3:00, 5:00, 6:00, 8:00, 16:00, 17:00, 20:00, 21:00, 23:00 et al. n=55 |
| **C(24,13)** | 25.71 | 26.83 | 22.22 | 45.00 | 88.57 | 73.17 | 66.67 | 85.00 | 0:00, 1:00, 2:00, 3:00, 4:00, 5:00, 6:00, 8:00, 16:00, 17:00, 20:00, 21:00, 23:00 et al. n=280 |
| **C(24,14)** | 28.57 | 31.71 | 25.93 | 50.00 | 88.57 | 73.17 | 66.67 | 85.00 | 0:00, 1:00, 2:00, 3:00, 4:00, 5:00, 6:00, 7:00, 8:00, 16:00, 17:00, 20:00, 21:00, 23:00 et al. n=875 |
| **C(24,15)** | 31.43 | 34.15 | 29.63 | 53.33 | 88.57 | 73.17 | 66.67 | 85.00 | 0:00, 1:00, 2:00, 3:00, 4:00, 5:00, 6:00, 7:00, 8:00, 9:00, 16:00, 17:00, 20:00, 21:00, 23:00 et al. n=1876 |
| **C(24,16)** | 37.14 | 36.59 | 33.33 | 58.33 | 88.57 | 73.17 | 66.67 | 85.00 | 0:00, 1:00, 2:00, 3:00, 4:00, 5:00, 6:00, 7:00, 8:00, 9:00, 10:00, 16:00, 17:00, 20:00, 21:00, 23:00 et al. n=2919 |
| **C(24,17)** | 45.71 | 41.46 | 40.74 | 61.67 | 88.57 | 73.17 | 66.67 | 85.00 | 0:00, 1:00, 2:00, 3:00, 4:00, 5:00, 6:00, 7:00, 8:00, 9:00, 10:00, 11:00, 16:00, 17:00, 20:00, 21:00, 23:00 et al. n= 3396 |
| **C(24,18)** | 57.14 | 46.34 | 44.44 | 66.67 | 88.57 | 73.17 | 66.67 | 85.00 | 0:00, 1:00, 2:00, 3:00, 4:00, 5:00, 6:00, 7:00, 8:00, 9:00, 10:00, 11:00, 12:00, 16:00, 17:00, 20:00, 21:00, 23:00 et al. n=2994 |
| **C(24,19)** | 62.86 | 48.78 | 48.15 | 71.67 | 88.57 | 73.17 | 66.67 | 85.00 | 0:00, 1:00, 2:00, 3:00, 4:00, 5:00, 6:00, 7:00, 8:00, 9:00, 10:00, 11:00, 12:00, 13:00, 16:00, 17:00, 20:00, 21:00, 23:00 et al. n=2001 |
| **C(24,20)** | 65.71 | 53.66 | 51.85 | 75.00 | 88.57 | 73.17 | 66.67 | 85.00 | 0:00, 1:00, 2:00, 3:00, 4:00, 5:00, 6:00, 7:00, 8:00, 9:00, 10:00, 11:00, 12:00, 13:00, 14:00, 16:00, 17:00, 20:00, 21:00, 23:00 et al. n=1001 |
| **C(24,21)** | 68.57 | 56.10 | 55.56 | 76.67 | 88.57 | 73.17 | 66.67 | 85.00 | 0:00, 1:00, 2:00, 3:00, 4:00, 5:00, 6:00, 7:00, 8:00, 9:00, 10:00, 11:00, 12:00, 13:00, 14:00, 15:00, 16:00, 17:00, 20:00, 21:00, 23:00 et al. n=364 |
| **C(24,22)** | 74.29 | 65.85 | 59.26 | 78.33 | 88.57 | 73.17 | 66.67 | 85.00 | 0:00, 1:00, 2:00, 3:00, 4:00, 5:00, 6:00, 7:00, 8:00, 9:00, 10:00, 11:00, 12:00, 13:00, 14:00, 15:00, 16:00, 17:00, 18:00, 20:00, 21:00, 23:00 et al. n=91 |
| **C(24,23)** | 80.00 | 70.73 | 62.96 | 81.67 | 88.57 | 73.17 | 66.67 | 85.00 | 0:00, 1:00, 2:00, 3:00, 4:00, 5:00, 6:00, 7:00, 8:00, 9:00, 10:00, 11:00, 12:00, 13:00, 14:00, 15:00, 16:00, 17:00, 18:00, 19:00, 20:00, 21:00, 23:00 et al. n=14 |
| **Hourly** | 88.57 | 73.17 | 66.67 | 85.0 | 88.57 | 73.17 | 66.67 | 85.0 | - |

C (24, r): selecting r time points from the 24 hours in one day.
